# Prehypertension and incidence of cardiovascular disease: a meta-analysis

**DOI:** 10.1186/1741-7015-11-177

**Published:** 2013-08-02

**Authors:** Yuli Huang, Sheng Wang, Xiaoyan Cai, Weiyi Mai, Yunzhao Hu, Hongfeng Tang, Dingli Xu

**Affiliations:** 1 Department of Cardiology, Nanfang Hospital, Southern Medical University, 1838 North Guangzhou Avenue, Guangzhou 510515, China; 2 Clinical Medicine Research Center, the Affiliated Hospital at Shunde, Southern Medical University (the First People’s Hospital of Shunde), Foshan, China; 3 Department of Cardiology, the First Affiliated Hospital of Sun Yat-sen University, Guangzhou, China

**Keywords:** Prehypertension, Cardiovascular diseases, Morbidity, Meta-analysis

## Abstract

**Background:**

Prospective cohort studies of prehypertension and the incidence of cardiovascular disease (CVD) are controversial after adjusting for other cardiovascular risk factors. This meta-analysis evaluated the association between prehypertension and CVD morbidity.

**Methods:**

Databases (PubMed, EMBASE and the Cochrane Library) and conference proceedings were searched for prospective cohort studies with data on prehypertension and cardiovascular morbidity. Two independent reviewers assessed the reports and extracted data. The relative risks (RRs) of CVD, coronary heart disease (CHD) and stroke morbidity were calculated and reported with 95% confidence intervals (95% CIs). Subgroup analyses were conducted on blood pressure, age, gender, ethnicity, follow-up duration, number of participants and study quality.

**Results:**

Pooled data included the results from 468,561 participants from 18 prospective cohort studies. Prehypertension elevated the risks of CVD (RR = 1.55; 95% CI = 1.41 to 1.71); CHD (RR = 1.50; 95% CI = 1.30 to 1.74); and stroke (RR = 1.71; 95% CI = 1.55 to 1.89). In the subgroup analyses, even for low-range prehypertension, the risk of CVD was significantly higher than for optimal BP (RR = 1.46, 95% CI = 1.32 to 1.62), and further increased with high-range prehypertension (RR = 1.80, 95% CI = 1.41 to 2.31). The relative risk was significantly higher in the high-range prehypertensive populations than in the low-range populations (χ^2^*=* 5.69, *P =* 0.02). There were no significant differences among the other subgroup analyses (*P*>0.05).

**Conclusions:**

Prehypertension, even in the low range, elevates the risk of CVD after adjusting for multiple cardiovascular risk factors.

## Background

In 2003, the seventh report of the Joint National Committee on Prevention, Detection, Evaluation, and Treatment of High Blood Pressure (JNC 7) proposed a new blood pressure (BP) category of 120 to 139 mm Hg systolic blood pressure (SBP) or 80 to 89 mm Hg diastolic blood pressure (DBP) and designated it as “prehypertension” [1]. This proposal was based, at least in part, on a meta-analysis of 61 prospective studies, which indicated that mortality from ischemic heart disease and stroke in individuals aged 40 to 89 years increases in a log-linear relationship with BP, from levels as low as 115 mm Hg systolic and 75 mm Hg diastolic [1,2].

Since the JNC 7 proposal, epidemiologic studies have shown that prehypertension is a common worldwide condition in up to 30 to 50% of the studied population [3,4]. Approximately 90% of individuals with prehypertension have at least one other cardiovascular risk factor and 68% have at least one significant clinical risk factor for heart disease or stroke [5]. Some studies have demonstrated that prehypertension is an independent risk factor for cardiovascular disease (CVD) [6-9], while others have not shown the same results after data were adjusted for baseline cardiovascular risk factors [10,11]. It remains unclear whether mild BP elevation directly increases the risk of cardiovascular disease or whether other concurrent risk factors are responsible for the increase [12]. Furthermore, arguments against using the term “prehypertension” also include the fact that there is heterogeneity within this category, as the risk of progressing to hypertension and developing CVD is higher in individuals with BP 130 to 139/85 to 89 mm Hg than in those with BP 120 to 129/80 to 84 mm Hg [3,13].

Given these inconsistent results, a meta-analysis of prospective cohort studies that examines the association of prehypertension with CVD morbidity may help clarify this issue. The objective of the present study was to evaluate the association between prehypertension and composite CVD, coronary heart disease (CHD) and stroke incidence.

## Methods

### Search strategy and selection criteria

We searched the electronic databases (PubMed, EMBASE and the Cochrane Library) up to the third week of December 2012 using the search terms: “prehypertension”, “prehypertensive”, “pre-hypertension”, “pre-hypertensive”, “high-normal blood pressure”, “high normal blood pressure”, “optimal blood pressure”, “borderline hypertension” or “borderline blood pressure”, and “cardiovascular disease”, “cardiovascular events”, “coronary artery disease”, “coronary heart disease”, “ischemic heart disease”, “stroke” or “cerebrovascular disease”. We restricted the search to human studies. Terms were explored whenever possible within each database. There were no language or publication form restrictions. Conference proceedings for the past 10 years from the American College of Cardiology Meeting, American Heart Association Scientific Sessions and the European Society of Cardiology Congress, and the reference lists of potentially relevant studies were also searched manually.

Studies were included if they met the following criteria: (1) prospective cohort studies of participants aged ≥18 years; (2) BP and other cardiovascular risk factors were evaluated at baseline; (3) the follow-up duration was ≥2 years and the study assessed the incidence of composited CVD, CHD or stroke morbidity; (4) they reported the multivariate-adjusted relative risks (RRs, including study-specific relative risk ratios or hazard ratios) and 95% confidence intervals (CIs) for events associated with prehypertension (BP 120 to 139/80 to 89 mm Hg) *vs.* reference (optimal BP, BP <120/80 mm Hg) or reported RRs and 95% CIs of low-range (BP 120 to 129/80 to 84 mm Hg) and high-range prehypertension (BP 130 to 139/85 to 89 mm Hg) *vs.* reference, respectively.

Studies were excluded if: (1) enrollment depended on having a particular risk factor condition; (2) they reported only age- and gender-adjusted relative risk; and (3) data were derived from the same cohort or from secondary analysis, or from combined analysis of other cohort studies.

If duplicate studies were derived from the same cohort and offered the same outcome messages, the latest published study was included. However, if duplicate studies offered additional messages for subgroup analysis that could not be derived from the primary included study, they were included in the subgroup analysis.

### Data extraction and quality assessment

Two investigators worked independently (YH and XC) to identify potentially relevant articles using the search strategy defined earlier. Full manuscripts of potentially relevant studies were obtained and reviewed according to predefined criteria. Information on study and participant characteristics, follow-up duration, and outcome assessment was abstracted and transferred to specially designed, pretested forms. Discrepancies were resolved by discussion with other investigators (WM, SW). When the primary outcome data were unpublished, we contacted the principal author for additional information.

The quality of each study was evaluated with reference to the US Preventive Task Force guidelines and a modified checklist used in previous studies [14-16]. This checklist assessed the following eight characteristics: (1) prospective study design; (2) maintenance of comparable groups; (3) adequate adjustment of potential confounders (at least five of six factors: age; sex; diabetes mellitus (DM); body mass index (BMI) or other measure of overweight/obesity; cholesterol; and smoking); (4) documented loss to follow-up rate; (5) outcome assessed blind to baseline status; (6) clear definition of exposures (prehypertension) and outcomes; (7) temporality (BP measured at baseline, not at the time of outcomes assessment); and (8) follow-up duration ≥2 years. Studies were graded as good quality if they met 7 to 8 criteria, fair for 4 to 6 criteria, and poor for <4 criteria.

### Data synthesis and analysis

The primary outcome considered was the risk of composited CVD morbidity, and secondary outcomes were risks of CHD and stroke morbidity associated with prehypertension, respectively. Subgroup analyses of the primary outcome were conducted according to BP (low-range prehypertension *vs.* high-range prehypertension); participant’s age (average <55 years *vs.* ≥55 years); gender (men *vs.* women); ethnicity (Asians *vs.* non-Asians); follow-up duration (<10 years *vs.* ≥10 years); participant number (<10,000 *vs.* ≥10,000); and study quality (good (score 7 to 8) *vs.* fair (score 4 to 6)).

Study-specific risk ratios or hazard ratios were used as the common measure of association between prehypertension and CVD across studies. Multivariate-adjusted RRs and 95% CIs were used for analysis. We logarithmically transformed these values in every study and calculated the corresponding standard errors (SEs) to stabilize the variance and normalize the distribution [15,16]. The statistical analysis used the inverse variance approach to combine log relative risks and SEs. When multivariate-adjusted RRs and 95% CIs for events associated with prehypertension were available, these data were used directly in the pooled meta-analysis calculations. For studies that published the RRs and 95% CIs of specific subgroups (for example, men and women, low-range and high-range prehypertension, or age-specific subgroups), but did not report an estimated overall risk, the information for each subgroup was used to calculate the overall RRs and 95% CIs for entry into the meta-analysis calculations.

We used χ^2^ and I^2^ statistics to test heterogeneity (25%, 50% and 75% representing low, moderate and high heterogeneity, respectively) [17]. Fixed-effects models were used for comparison with random-effects models on the overall risks estimate and yielded similar findings, but we detected between-study heterogeneity for several outcomes; therefore, results from the random-effects models are presented here. To assess for publication bias, we constructed funnel plots for each outcome in which the ln (RR) was plotted against its SE. Additionally, we conducted sensitivity analyses in which the pooled RR was recalculated by omitting one study at a time. *P-*values were two-tailed and the statistical significance was set at 0.05. All analyses were performed with RevMan software (version 5.1 for Windows, The Cochrane Collaboration, Copenhagen, Denmark).

We also determined the population-attributable risk (PAR) for prehypertension on the basis of the pooled RR. PAR% expresses the proportion of disease in the study population that is attributable to the exposure (prehypertension) and could be eliminated if the exposure was eliminated. The PAR% was calculated as PAR% = (Pe)(RR - 1)/((Pe)(RR - 1) + 1)) × 100, where Pe is the proportion of the population exposed to the risk factor (prehypertension), and RR indicates multivariate-adjusted relative risk [9].

## Results

### Selected studies and characteristics

**Figure 1:**
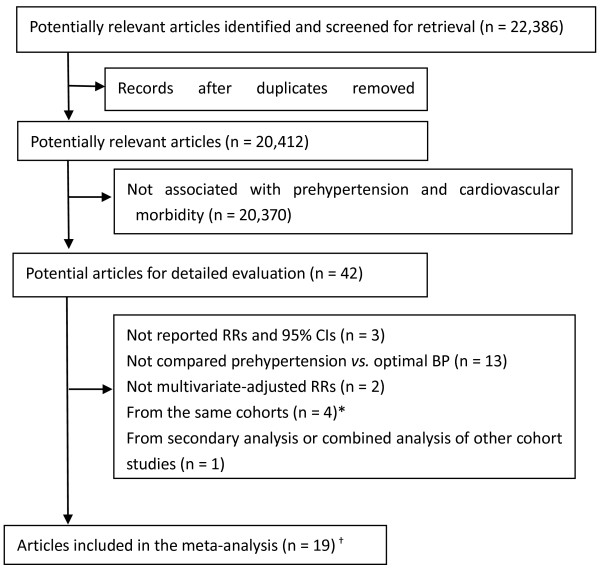
**Flow of selection for studies through review.** BP, blood pressure; CIs, confidence intervals; RRs indicates relative risks.^*^ Only the latest of the published duplicate studies from the same cohort was included if they offered the same outcome messages. However, one of these studies offered additional messages for subgroup analysis according to BP and gender [6], which could not be derived from the primary included study [9], so it was re-included when performing the subgroup analyses. ^†^ Data were derived from 18 prospective cohort studies (two articles were from the Strong Heart Study and reported the risk factors for CHD [19] and stroke [24], respectively).

The selection of studies for inclusion in the meta-analysis is shown in Figure 1. Of the initial 22,386 records, two reviewers determined independently that 42 required a review of the full manuscript. Our final primary analysis included 19 articles [7-11,18-31], with a total of 468,561 participants, derived from 18 prospective cohort studies (two articles were from the Strong Heart Study and reported the risk factors for CHD [19] and stroke [24], respectively). Eighteen of the primary papers were published in full and one was in abstract form [10]. However, study data from this abstract were acquired by correspondence with the main author. One article from the Framingham Heart Study was excluded for primary outcome analysis, because more recent data from the same cohort were available [9]. However, as this article offered additional messages for subgroup analyses according to BP and gender that could not be derived from the article included in the primary group [9], the study data were re-entered for subgroup analyses. Table 1 summarizes the key characteristics of the included studies. All prospective cohort studies were derived from the general population. Of the 18 studies 11 were from Asia (3 from China [7,25,29], 6 from Japan [18,23,26-28,31], and 2 from Iran [10,30]); 5 were from the United States [8,9,19-21,24]; and 1 each was from Turkey [22] and Germany [11]. The proportion of Asians was 79.6% (n = 372,927).

**Table 1:** Study characteristics

**Study**	**Country**	**Prevalence of prehypertension (%)**	**Sample size (% women)**	**Age (y), average (range or SD)**	**Follow-up (y)**	**Participants with baseline CVD excluded**	**Events for analysis**
Wu, 2002^*^[7]	China	35.3	27,739 (46.3)	47.4 (35 to 64)	7	Not report	CVD
Asayama, 2004 [18]	Japan	46	1,702 (61)	60.6 (10.7)	10.6	Free of stroke	Stroke
Liszka, 2005 [8]	United States	33	8,986 (54.6)	NA (≥25)	18	No	CVD, stroke, MI
Lee, 2006 [19]	United States	32.6	4,372 (60.6)	56.2(45 to 74)	12	Yes	CHD
Qureshi, 2005 [9]	United States	41.1	5,181 (55.3)	44.0 (8.6)	31	Yes	CAD, stroke, MI
Kshirsagar,2006 [20]	United States	37.3	8,960 (55)	53(45 to 64)	11.6	Yes	CVD, CHD, stroke
Hsia, 2007 [21]	United States	39	60,785 (100)	62.8 (7.0)	7.7	Yes	CVD, MI, stroke
Onat, 2008 [22]	Turkey	32.8	3,034 (50.4)	48 (12)	6.6	Free of DM and CHD	CHD
Kokubo, 2008 [23]	Japan	35	5,494 (53)	55 (30 to 79)	11.7	Yes	CVD, MI, stroke
Zhang, 2008 [24]	United States	32.1	4,507 (60)	56 (45 to 74)	13.4	Free of stroke	Stroke
Gu, 2009 [25]	China	34.5	158,666 (51)	56 (≥40)	7.7	No	CVD, CHD, stroke
Ikeda, 2009 [26]	Japan	43	33,372 (65)	54 (40 to 69)	11.0	Yes	CHD, stroke
Ishikawa, 2010 [27]	Japan	32.3	11,000 (61.3)	55.1 (11.5)	10.7	Yes	CVD
Tanaka, 2010 [28]	Japan	25.2	22,676 (66)	62 (40 to 80)	2.7	Yes	Ischemic stroke
Wu, 2012 [29]	China	30.0	100,116 (20.1)	49.4 (30 to 70)	4	Yes	CVD, stroke
Hadaegh, 2013 [30]	Iran	34.5	6,273(57)	47.1 (≥30)	9.3	Yes	CVD, CHD
Sadeghi, 2012^†^[10]	Iran	36	3,255 (100)	49.7 (≥35)	6.7	Yes	CVD, CHD, stroke
Fukuhara, 2012 [31]	Japan	37.7	2,634 (58)	59.1 (≥40)	19	Yes	CVD, CHD, Stroke
Erbel, 2012 [11]	Germany	26.2	4,181 (53)	59.3 (40 to 75)	7.18	Yes	MI,stroke , revascularization

^*^ Article in Chinese; ^†^ Authors contacted for clarification of data.

*CHD* Coronary heart disease, *CVD* Cardiovascular disease, *DM* Diabetes mellitus, *MI* Myocardial infarction, *NA* indicates not available.

The prehypertension incidence ranged from 25.2% [28] to 46.0% [18]. The sample size ranged from 1,702 [18] to 158,666 [25]. The follow-up duration ranged from 2.7 years [28] to 31 years [9]. Two studies enrolled women only [10,21], whereas all others enrolled both genders. All studies adjusted adequately for potential confounders (at least five of six factors: age, sex, DM, BMI or other measure of overweight/obesity, cholesterol and smoking) except for one study that adjusted for age, sex, heart rate, smoking and obesity [22]. Thirteen studies were graded as good quality and five were graded as fair quality. The details of the quality assessment and adjusted confounders are presented in Additional file 1: Table S1.

### Primary and secondary outcomes

**Figure 2:**
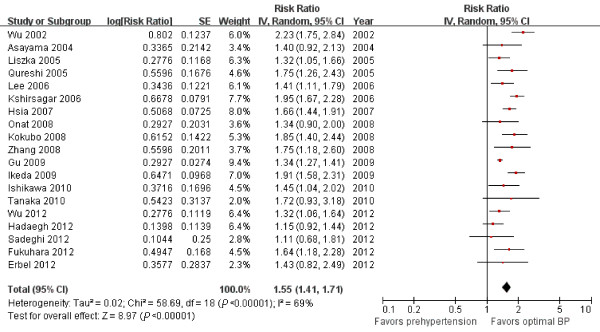
Forest plot of comparison: prehypertension vs. optimal blood pressure, outcome: cardiovascular morbidity.

The data were heterogeneous (I^2^ = 69%), so we used the random-effects model to combine results from all studies. In this model, the presence of prehypertension was associated with a 55% increase in CVD morbidity after multivariate adjustment for established cardiovascular risk factors (RR = 1.55; 95% CI = 1.41 to 1.71, *P*<0.00001, Figure 2). A visual inspection of the funnel plot found no evidence of publication bias (Additional file 1: Figure S1).

**Figure 3:**
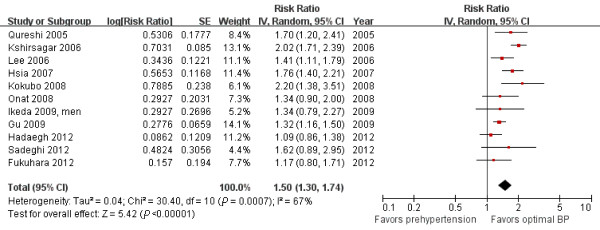
Forest plot of comparison: prehypertension vs. optimal blood pressure, outcome: coronary heart disease.

**Figure 4:**
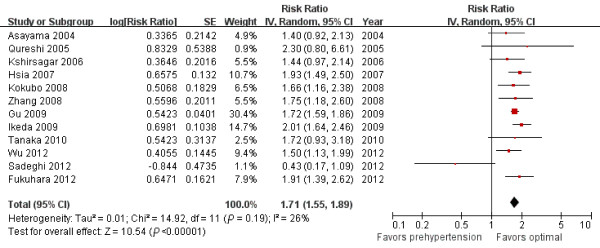
Forest plot of comparison: prehypertension vs. optimal blood pressure, outcome: stroke.

Eleven (n = 292,026) and 12 studies (n = 406,539) reported multivariate-adjusted RRs and 95% CIs for CHD and stroke associated with prehypertension, respectively. We used the random-effects model on the pooled data from these studies and calculated a 50% increase in CHD incidence (RR = 1.50; 95% CI = 1.30 to 1.74, *P <*0.00001, I^2^ = 67%, Figure 3) and a 71% increase in stroke incidence (RR = 1.71; 95% CI = 1.55 to 1.89, *P <*0.00001, I^2^ = 26%, Figure 4). However, the difference between the incidence of CHD and stroke was not significant (χ^2^*=* 2.13, *P =* 0.14).

On the basis of the pooled RR, the PARs of CVD, CHD and stroke for prehypertension were 15.9%, 14.6% and 19.6%, respectively.

### Subgroup analyses

In the subgroup analyses, prehypertension significantly predicted higher CVD risk across subgroups with analyses conducted according to participant’s age, gender, ethnicity, follow-up duration, participant number and study quality. Even low-range prehypertension increased the risk of CVD compared to optimal BP (RR = 1.46, 95% CI = 1.32 to 1.62), and the risk further increased with high-range prehypertension (RR = 1.80, 95% CI = 1.41 to 2.31). The relative risk was higher in the high-range than in the low-range prehypertensive populations (χ^2^*=* 5.69, *P =* 0.02, Table 2). We found no significant differences in the other subgroups (Table 2).

**Table 2:** Subgroup analyses of the association between prehypertension and cardiovascular morbidity

**Subgroup**	**Risk ratio (95% CI)**	***P-*value between subgroups**
**Blood pressure range**		
Low-range prehypertension	1.46 (1.32, 1.62)	0.02
High-range prehypertension	1.63 (1.47, 1.80)	
**Gender**		
Men	1.80 (1.44, 2.24)	0.11
Women	1.46 (1.28, 1.67)	
**Race/ethnicity**		
Asian	1.54 (1.34, 1.77)	0.96
Non-Asian	1.55 (1.36, 1.77)	
**Participant’s average age**		
<55 years	1.55 (1.31, 1.84)	0.72
≥55 years	1.50 (1.35, 1.67)	
**Follow-up duration**		
<10 years	1.45 (1.27, 1.67)	0.21
≥10 years)	1.63 (1.45, 1.84)	
**Participant number**		
<10,000	1.59 (1.38, 1.84)	0.52
≥10,000	1.49 (1.28, 1.73)	
**Study quality**		
Good (score 7 to 8)	1.54 (1.37, 1.73)	1
Fair (score 4 to 6)	1.54 (1.21, 1.96)	

### Sensitivity analyses

Multiple methods were used to test the sensitivity and the primary results were not influenced by the use of fixed-effect models compared with random-effect models, odds ratios compared with RRs or recalculation by omitting one study at a time.

## Discussion

This meta-analysis found, after controlling for multiple cardiovascular risk factors, a robust and significant association between prehypertension and CVD incidence. The results were consistent across age, gender, trial characteristics, follow-up duration and ethnicity. More importantly, even low-range prehypertension increased the risk of CVD compared with optimal BP and the risk was higher with high-range prehypertension. The PARs calculation indicated 15.9% of CVD, 14.6% of CHD and 19.6% of stroke cases could be prevented if prehypertension was eliminated.

The primary strength of this meta-analysis was that the included studies were restricted to prospective cohort studies only and they reported multivariate-adjusted relative risks. It has been reported that prehypertension is associated with other cardiovascular risk factors [3,32,33]. In several multivariate analyses, high BMI was the strongest predictor of prehypertension among traditional risk factors [4,34,35]. In large populations, individuals with prehypertension are also more likely to have diabetes [5], impaired fasting glucose [4], metabolic syndrome [36], and dyslipidemia than normotensive individuals [4]. After controlling for these risk factors, some prospective studies have demonstrated prehypertension is still an independent risk factor for CVD [6-9], while others have not shown the same results [10,11]. In our meta-analysis, all of the included studies adequately adjusted for potential confounders (at least five of six factors: age, sex, DM, BMI or other measure of over-weight/obesity, cholesterol and smoking) except one study which adjusted for age, sex, heart rate, smoking and obesity [22]. This feature probably mitigated the possibility of known confounders influencing the association between prehypertension and CVD.

The term “prehypertension” has been contentious since the JNC 7 proposal [37]. Other national and international hypertension guidelines have adopted neither the term nor the concept behind prehypertension, preferring to retain the older classification systems for BP [3]. For example, the 2007 report from the Task Force for the Management of Arterial Hypertension of the European Society of Hypertension (ESH) and of the European Society of Cardiology (ESC) preferred to term the 120 to 129/80 to 84 mm Hg group as “normal blood pressure” and the 130 to 139/85 to 89 mm Hg group as “high normal” [38].

One of the most important arguments against the term “prehypertension” is that the risks of progressing to hypertension and developing cardiovascular events are different in those with BP 130 to 139/85 to 89 mm Hg than in those with BP in the 120 to 129/80 to 84 mm Hg range. Our meta-analysis reported that even low-range prehypertension increased the risk of composited CVD compared with optimal BP and the risk was higher with high-range prehypertension. In a recently published meta-analysis, Lee *et al*. reported that prehypertension was associated with a higher risk of incident stroke [16]; however, the association of low-range prehypertension and stroke was not significant (RR = 1.22, 95% CI = 0.95 to 1.57, *P* = 0.11). Another recently published meta-analysis by Shen *et al*. had reported that prehypertension was associated with a higher risk of CHD; however, the association of low-range prehypertension and CHD was not significant [39]. In contrast, our analysis found that even low-range prehypertension increases the risk of CVD. One possible cause of these inconsistent findings may be the differences in the events assessed. Lee’s and Shen’s analyses focused on stroke and CHD, respectively [16,39]. In contrast, our analysis focused primarily on composited cardiovascular morbidity. Also, we used a wider search strategy with more search terms, including “prehypertension”, “prehypertensive”, “high normal blood pressure”, “optimal blood pressure”, “borderline hypertension” or “borderline blood pressure”. We believe that the wider search strategy is important for meta-analyses to avoid missing potentially relevant studies. Meta-analyses may be biased when the literature search fails to identify all relevant studies.

Our analysis is supported by a study by Arima *et al*. [40], which included 346,570 participants from 36 cohort studies in the Asia-Pacific region, showing that after adjusting for age, sex, cholesterol and smoking, the hazard ratio for CVD was 1.41 (95% CI = 1.31 to 1.53) in prehypertension. However, there are some important differences in the two analyses. First, most of our included studies were adequately adjusted for potential confounders, including BMI and DM, which were the strongest predictors of prehypertension [4,34,35]. However, BMI and DM were not adjusted in Arima’s analysis [40]. Second, our analysis used worldwide data, while Arima *et al*. used data only from the Asia-Pacific region. Our subgroup analysis found no difference between Asians and non-Asians.

Considering that the great incidence of prehypertension is up to 30 to 50% [3,4], successful intervention in such a large population could, therefore, have a major public health impact. An effective massive public health intervention may be chiefly educational aiming both at patients and physicians. Healthcare professionals should recommend lifestyle changes early to subjects with prehypertension. However, since the incidence of CVD increased across the whole range of prehypertension, physicians should be aware of which subgroup of the population are at high risk for CVD and of steps that should be taken to treat modifiable risk factors in these people, especially in high-range prehypertension [41]. It had been reported that many risk factors, including overweight, dyslipidemia and impaired glucose metabolism were associated with prehypertension and adverse events [33,42,43]. These associated CV risk factors are indicators for selection of subpopulations for future controlled trials of pharmacological treatment, and controlling these factors is helpful in clinical management of prehypertension [44].

This meta-analysis has some limitations. First, we had no access to individual patient-level data. However, as discussed previously, most of the included studies were adequately adjusted for potential confounding risk factors and were of good quality; this may have mitigated the possibility of other cardiovascular risk factors influencing the association of prehypertension and CVD. Second, in most included studies, the determination of prehypertension was based on one single-day measurement, albeit with multiple readings. This may misclassify BP levels due to “white coat effect” or “masked hypertension” and lead to a dilution bias. However, our results are indicative, on the basis of a “snapshot” BP measurement, that prehypertension is associated with increased CVD risk. Finally, selection and publication bias are always possible. We used multiple assessors to minimize the likelihood of such bias, including a comprehensive search strategy, two independent reviewers, standardized eligibility criteria, and funnel plot testing for assessment of publication bias. We consider it unlikely that the results and our conclusions were influenced by such bias.

## Conclusions

Prehypertension, even at low levels, is associated with a high risk of CVD. This reaffirms the importance of the definition of prehypertension and its importance to health professionals engaged in the primary prevention of CVD. However, because of the significant difference in the risk of CVD for BP between 120 to 129/80 to 84 mm Hg and 130 to 139/85 to 89 mm Hg, we suggest that this category should be subdivided into low- and high-range prehypertension and that lifestyle modification should be advocated earlier than usual in prehypertension. Further studies are needed to reveal better predictors of high-risk subpopulations with prehypertension (especially in high-range prehypertension) to select subpopulations for future controlled trials of pharmacological treatment.

## Abbreviations

BMI: Body mass index; BP: Blood pressure; CHD: Coronary heart disease; CIs: Confidence intervals; CVD: Cardiovascular disease; DBP: Diastolic blood pressure; DM: Diabetes mellitus; ESC: European Society of Cardiology; ESH: European Society of Hypertension; JNCD 7: The seventh report of the Joint National Committee on Prevention, Detection, Evaluation, and Treatment of High Blood Pressure; PAR: Population-attributable risk; RRs: Relative risks; SBP: Systolic blood pressure; SEs: Standard errors.

## Competing interests

The authors declare that they have no competing interests.

## Authors’ contributions

YH, SW and DX conceived and designed the review. YH and XC identified and acquired reports of trials, abstracted data and assessed risk of bias. YH, XC and WM drafted the manuscript. HT and YH provided supervision. YH and HT also conducted the statistical analyses and contacted authors of included studies to obtain additional information. All of the authors contributed to the interpretation of data and all of the authors critically revised the manuscript. All of the authors approved the final version of the manuscript submitted for publication and are guarantors for the study.

## Pre-publication history

The pre-publication history for this paper can be accessed here:

http://www.biomedcentral.com/1741-7015/11/177/prepub

## Supplementary Material

Additional file 1: Table S1 Quality assessment and confounders adjusted in the included studies. **Figure S1.** Funnel plot of comparison, prehypertension vs. optimal blood pressure, outcome: cardiovascular morbidity. Click here for file
