# Supplementary material for: Prehypertension and incidence of cardiovascular disease: a meta-analysis
Source: BMC Med. 2013 Aug 2;11:177. doi: 10.1186/1741-7015-11-177 (PMC3750349; doi:10.1186/1741-7015-11-177)
Supplement: Additional file 1: Table S1 — Quality assessment and confounders adjusted in the included studies. Figure S1. Funnel plot of comparison, prehypertension vs. optimal blood pressure, outcome: cardiovascular morbidity. [file 1741-7015-11-177-S1.doc]

**Additional file 1: Table S1. Quality Assessment and Confounders Adjusted in the Included Studies**

| Study | Risk factors adjusted | Study quality |
| --- | --- | --- |
| Wu, 2002^*^ [7] | Age, sex, BMI, DM, CHOL and smoking | Fair |
| Asayama, 2004 [18] | Age, sex, DM, hypercholesterolemia, smoking and history of CVD | Good |
| Liszka, 2005 [8] | Age, race, sex, smoking, BMI, exercise, CHOL, DM, and history of HF, MI, and stroke | Fair |
| Lee, 2006 [19] | Age, sex, DM, smoking, LDL-C, HDL-C and albuminuria | Good |
| Qureshi, 2005 [9] | Age, sex, smoking, obesity, DM, hypercholesterolemia, and study period. | Good |
| Kshirsagar,2006 [20] | Age, race, sex, BMI, DM, smoking, LDL-C, HDL-C, education level, sport index, CHOL lowering medication, fibrinogen, vWF and WBC. | Good |
| Hsia, 2007 [21] | Age, BMI, DM, CHOL, and smoking. | Good |
| Onat, 2008 [22] | Age, sex, heart rate, smoking, obesity | Fair |
| Kokubo, 2008 [23] | Age, BMI, hyperlipidemia, DM, smoking and drinking status. | Good |
| Zhang, 2008 [24] | Age, sex, BMI, DM, waist circumference, TG, LDL-C, HDL-C, physical activity, smoking and drinking status and microalbuminuria | Good |
| Gu, 2009 [25] | Age, sex, education level, smoking and drinking status, BMI, physical activity, antihypertensive medication, history of CVD or DM, geographic region and urbanization | Good |
| Ikeda, 2009 [26] | Age, sex, BMI, smoking and drinking status, antihypertensive medication, history of DM, CHOL, and public health center areas. | Good |
| Ishikawa, 2010 [27] | Age, sex, BMI, hyperlipidemia, DM, smoking and drinking status | Good |
| Tanaka 2010 [28] | Sex, age, CHOL, HDL-C, renal dysfunction, BMI, DM, smoking and drinking status and atrial fibrillation | Fair |
| Wu, 2012 [29] | Age, sex, TG, CHOL, LDL-C, HDL-C, FBG, UA, BMI, smoking and drinking status | Fair |
| Hadaegh, 2013 [30] | Age, sex, CHOL, BMI, smoking, DM, lipid-lowering medication and family history of premature CVD | Good |
| Sadeghi 2012^†^ [10] | Age, DM, CHOL, BMI, smoking and menopause | Good |
| Fukuhara, 2012 [31] | Age, sex, BMI, CHOL, HDL-C, DM, chronic kidney disease, electrocardiogram abnormalities, smoking, drinking, and regular exercise. | Good |
| Erbel, 2012 [11] | Age, sex, cholesterol, DM, and smoking | Good |

^*^ Article in Chinese; ^†^ Authors contacted for clarification of data

BMI indicates body mass index; DM: diabetes mellitus; CHOL: cholesterol level; CVD: cardiovascular disease; HF: heart failure; MI: myocardial infarction; LDL-C: low-density lipoprotein cholesterol; HDL-C: high-density lipoprotein cholesterol; vWF: von Willebrand factor; WBC: white blood cell count; TG: triglyceride; FBG: fasting blood glucose; UA: serum uric acid.


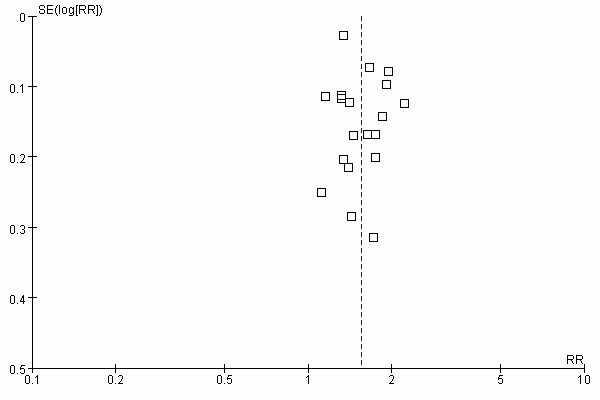


**Additional file 1: Figure S1.** Funnel plot of comparison, prehypertension vs. optimal blood pressure, outcome: cardiovascular morbidity.
